# Field Performance of Transgenic Sugarcane Lines Resistant to *Sugarcane Mosaic Virus*

**DOI:** 10.3389/fpls.2017.00104

**Published:** 2017-02-08

**Authors:** Wei Yao, Miaohong Ruan, Lifang Qin, Chuanyu Yang, Rukai Chen, Baoshan Chen, Muqing Zhang

**Affiliations:** ^1^State Key Laboratory of Conservation and Utilization of Subtropical Agri-Biological Resources, Guangxi UniversityNanning, China; ^2^Key Laboratory of Sugarcane Biology and Genetic Breeding, Ministry of Agriculture, Fujian Agriculture and Forestry UniversityFuzhou, China; ^3^IRREC-IFAS, University of FloridaFort Pierce, FL, USA

**Keywords:** sugarcane, field evaluation, *sugarcane mosaic virus*, transgene, virus resistance, coat protein

## Abstract

Sugarcane mosaic disease is mainly caused by the *sugarcane mosaic virus* (SCMV), which can significantly reduce stalk yield and sucrose content of sugarcane in the field. Coat protein mediated protection (CPMP) is an effective strategy to improve virus resistance. A 2-year field study was conducted to compare five independent transgenic sugarcane lines carrying the SCMV-CP gene (i.e., B2, B36, B38, B48, and B51) with the wild-type parental clone Badila (WT). Agronomic performance, resistance to SCMV infection, and transgene stability were evaluated and compared with the wild-type parental clone Badila (WT) at four experimental locations in China across two successive seasons, i.e., plant cane (PC) and 1st ratoon cane (1R). All transgenic lines derived from Badila had significantly greater tons of cane per hectare (TCH) and tons of sucrose per hectare (TSH) as well as lower SCMV disease incidence than those from Badila in the PC and 1R crops. The transgenic line B48 was highly resistant to SCMV with less than 3% incidence of infection. The recovery phenotype of transgenic line B36 was infected soon after virus inoculation, but the subsequent leaves showed no symptoms of infection. Most control plants developed symptoms that persisted and spread throughout the plant with more than 50% incidence. B48 recorded an average of 102.72 t/ha, which was 67.2% more than that for Badila. The expression of the transgene was stable over many generations with vegetative propagation. These results show that SCMV-resistant transgenic lines derived from Badila can provide resistant germplasm for sugarcane breeding and can also be used to study virus resistance mechanisms. This is the first report on the development and field performance of transgenic sugarcane plants that are resistant to SCMV infection in China.

## Introduction

Sugarcane (*Saccharum officinarum L*.) accounts for 90% of all sugar produced in China and 70% of sugar production worldwide. In 2015, 10.6 million metric tons (MMT) of sugar were produced in China (Sugar: World Markets and Trade, 2015, http://www.fas.usda.gov/data/ sugar-world-markets-and-trade). Sugarcane is also an important industrial crops for sugar industries and allied industries that produce alcohol, acetic acid, butanol, paper, plywood, industrial enzymes, and animal feed (Lakshmanan et al., 2005; McQualter et al., 2005; Petrasovits et al., 2013; Raghavi et al., 2015; Zhang et al., 2015a).

Sugarcane mosaic disease is a serious viral disease that affects sugarcane and significantly reduces yields in terms of tons of cane per hectare (TCH) and tons of sucrose per hectare (TSH). The sugarcane mosaic virus subgroups of potyvirus, sugarcane mosaic virus (SCMV), or sorghum mosaic virus (SrMV) are the causative agents of mosaic disease in sugarcane (Gao et al., 2011). Potyviruses are positive sense single stranded RNA viruses with a ~10 kb genome and ORFs that encode 10 mature functional proteins, including P_1_, HC-Pro, P_3_, 6K_1_, CI, 6K_2_, VPg, NIa-Pro, Nib, and CP. The virus damages chloroplasts and blocks photosynthesis, which decreases photosynthetic products and diminishes TCH and TSH levels (Xu et al., 2008; Chauhan et al., 2015). Coat protein-mediated protection (CPMP) strategies are effective in improving resistance to plant viruses. Indeed, virus-resistant transgenic papaya, squash, and potato have been commercially released in the United States (Tepfer, 2002; Tecson et al., 2008), while over-expression of the coat protein (CP) gene from the alfalfa mosaic virus (AMV) in transgenic pea, tomato, and tobacco plants produced varying degrees of virus resistance (Fitchen and Beachy, 1993; Timmerman-Vaughan et al., 2001). Transgenic white clover plants that express AMV-CP show meiotically stable virus resistance under both greenhouse and field conditions (Panter et al., 2012). One mechanism by which transgenic plants overexpressing viral CPs can manifest viral resistance is through the interference of surplus CP with viral processes, such as virion assembly and disassembly, as well as viral movement within the plant (Bendahmane et al., 2007; Mehta et al., 2013).

Multinational agribusinesses and sugar industries worldwide have recently been making significant investments in the development of transgenic sugarcane to meet the needs of expanding sugar and biofuel markets. In addition to viral resistance, these commercially available transgenic sugarcanes can also display drought tolerance and resistance to insects (e.g., stalk borer) and herbicides (Weng et al., 2011; Joyce et al., 2014). The commercial success of any transgenic sugarcane depends on the uniform and stable expression of the transformed trait(s) and agronomic performance that is comparable to elite commercial cultivars. Given the genetic variability that is inherent in transgenic sugarcane plants, field assessment of a large population of independent transgenic events is required to identify commercially valuable transgenic sugarcanes (Butterfield et al., 2002; Gilbert et al., 2005; Basnayake et al., 2012). However, only a few studies to date have evaluated the agronomic performance of transgenic sugarcane plants in the field (Guo et al., 2008; Gilbert et al., 2009; Weng et al., 2011; Basnayake et al., 2012; Joyce et al., 2014). In this study, we used microprojectile bombardment to produce transgenic sugarcane lines (Badila) that expressed the CP gene from the *sugarcane mosaic virus* E strain (SCMV-CP) and investigated the expression stability, agronomic performance, and yield characteristics of transgenic sugarcane plants in the field over a 2-year period at four different experimental locations in China.

## Materials and methods

### Sugarcane transformation and regeneration

**Figure 1:**
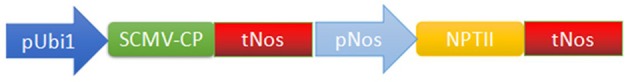
**Schematic illustration of the pUbi-SCMV-CP cassette**. pUbi1, promoter of maize ubiquitin gene; tNos, terminator of nopaline synthase gene; pNos, promoter of nopaline synthase gene; npt II, neomycin phosphotransferase II gene (kanamycin resistance for selection in plants).

The sugarcane (*cv*. Badila) obtained from the experimental field of the Fujian Agriculture and Forestry University was used as the explant in this study. Transformation with the construct pUbi-SCMV-CP (Figure 1) harboring the SCMV CP gene was achieved using a Bio-Rad 1000/He Biolistics gun at 1100 *psi* following the manufacturer's protocol (Bio-Rad, Hercules, CA). The pUbi-SCMV-CP construct was co-precipitated onto tungsten microprojectiles and transferred into precultured sugarcane embryogenic calli, followed by selection for kanamycin resistance and regeneration of transgenic sugarcane plants. The transformation and regeneration methods used have been previously described in detail (Yao et al., 2004; Zhang et al., 2015b). After selection on kanamycin containing media in tissue culture, transgenic sugarcane plants were grown in a greenhouse. Only one kanamycin-resistant regenerated plant was taken per single callus piece that emerged on selective medium. Consequently, each transgenic plant in this experiment came from an independent primary transformed cell, and thus multiple transgene integration sites in the genome of transgenic sugarcane plants could occur.

### Artificial inoculation of transgenic sugarcane plants

Transgenic sugarcane plants were vegetative propagated and grown in soil under greenhouse conditions for 6–8 weeks prior to inoculation. Viral inoculation was prepared as previously described by extracting crude sap from SCMV-infected sugarcane leaves using a mechanical sap extractor in 0.1 M phosphate buffer, pH 7.0 (Użarowska et al., 2009). Sugarcane plants were kept in the dark for 24 h before the surface of each leaflet was gently rubbed with an inoculum containing 2% (w/v) fine carborundum. Five days later, the second inoculation was administered to each plant to ensure inoculation effectiveness. Two weeks after the inoculation, the leaves of the inoculated sugarcane plants were examined every 2 days and scored for development of systemic symptoms. As a control, untransformed tissue cultures (TC) and the wild-type parent clone (WT) at the same growth age were inoculated using the same approach as the transgenic sugarcane plants. The reactions to artificial inoculation could be grouped into three different phenotypes as follows: (i) Susceptible type, wherein systemic symptoms were observed 14–21 days after inoculation (DAI); (ii) Recovery type, wherein systemic symptoms were observed at 14–21 DAI, but not on newly grown leaves at 28–42 DAI; and (iii) Resistant type, wherein plants were symptom free throughout their life cycles. SCMV-CP over-expression resulted in varying levels of virus resistance by transgenic sugarcane plants from Level 1 to Level 5, which were susceptible, resistant, moderately resistant, highly resistant, and immune, respectively, to SCMV.

### Field evaluation of transgenic sugarcane

The field evaluations reported here were conducted in accordance with a license for field release of specified GM sugarcane lines issued by the Management Office of China Agricultural GMO Safety (License NO.40/2006). The study was conducted over two successive seasons (2007 and 2008) at four different experimental field sites located at Fuzhou and Jianyan in the Fujian province as well as Yiwu and Wenling in the Zhejiang province. The field trial design consisted of 28 plots of transgenic sugarcane lines and non-transgenic control plants distributed in a one-hectare paddock in each experimental field site with 400 m^2^ (20∗20 m) and 4 replicates for each transgenic line and controls, respectively.

Five selected virus resistant transgenic sugarcane lines and WT plants from the first to fourth vegetative propagated generation were selected for the field evaluations. All sets of transgenic and non-transgenic control sugarcanes were cut into single bud setts. After sterilization with 5% carbendazim for 10 min, all single bud setts were planted in the ground in a randomized block design. Agronomic traits and field virus resistance of all sugarcane plants were evaluated through field trials as previously described (Gilbert et al., 2009). The field trials were performed as the commercial sugarcane production. Following the harvest, all transgenic sugarcane plants were destroyed in the field.

### Sugarcane yield traits and sucrose analyses

At harvest, agronomic parameters, including stalk diameter (~1 m above the base of the stalk), stalk height (from the base of the stalk to the first visible dewlap), stalk weight, millable stalk numbers, and length of internode and tons cane per hectare (TCH, tons/ha) were measured. Plant fresh weights were used to determine individual stalk weight (kg/stalk), and TCH was calculated as the product of stalk number and stalk weight. Sugarcane stalks were ground in a three-roller mill within 24 h of harvest to measure sucrose concentrations and cane fiber content, and the extracted juices were analyzed for Brix and Pol. Brix was determined using a refractometer to measure the soluble solid content of a sucrose-containing solution and Pol was tested using a saccharimeter to measure the apparent sucrose content of any substance (Arencibia et al., 1999; Gilbert et al., 2009).

### Selectable marker expression test

Expression of the *nptII* gene as a selectable marker was verified under field and greenhouse conditions using a kanamycin antibiotic assay (Gilbert et al., 2009). A 3 g/L solution of kanamycin monosulfate was mixed with 1 mL/L surfactant (Silwet L-77) and deionized water in a 1 L spray bottle. A stream of ~1 mL was sprayed onto young leaf whorls of 10 randomly selected stalks for each transgenic sugarcane line and non-transgenic control plant (WT and TC). Each sprayed stalk was marked with flagging labels for subsequent observation. Approximately 10 days later, each stalk was scored as either kanamycin susceptible (leaf chlorosis symptoms) or resistant (no leaf chlorosis), with kanamycin resistance indicating expression of the selectable *nptII* marker gene.

### Statistical analysis

Independent analyses were conducted for each of the agronomic traits of cane yield, internode length, stalk height, millable stalk number, single stalk weight, stalk diameter and Brix. The variance analysis was obtained using PROC GLM in SAS (SAS Institute, 1996). Years, locations, and replications were considered random effects, whereas genotype was considered a fixed effect. The performance of a transgenic line was regressed on the environmental index (deviation of the mean yield at that environment from the overall mean yield of all environments) as described by Eberhart and Russell (1966). In this model, the regression coefficient (bi) and the deviation from regression mean squares (${\text{S}}_{\text{d}}^{2}$) were considered as parameters of response and stability, respectively. Regression coefficients above 1.0 described genotypes with higher sensitivity to environmental change (below average stability) and greater specificity of adaptability to good environments. A regression coefficient below 1.0 provided a measurement of greater resistance to environmental change (above average stability), and thus increased the specificity of adaptability to the bad environments (Wachira et al., 2002; Rharrabti et al., 2003).

## Results

### Production of transgenic sugarcane plants expressing the SCMV-CP gene

The sugarcane cultivar Badila is highly susceptible to SCMV infection and displays excellent regeneration ability, and thus was selected as the parental line for transformation. The pUbi-SCMV-CP plasmid carrying the SCMV CP gene was transferred into Badila embryogenic calli *via* microprojectile bombardment. In 40 bombardment experiments, 2251 embryogenic calli were bombarded and 174 kanamycin resistant calli were obtained. After selective subculture and differentiation in the presence of antibiotic followed by root culture, 53 independent transgenic sugarcane seedlings were regenerated on selective medium containing 100 mg/L kanamycin. Of these, 13 sugarcane plants were positive for CP gene expression as verified by Southern blot (Yao et al., 2004) (Table 1). Among the 13 Southern blot positive transgenic lines tested, 5 transgenic lines scored Level 5, or 4 in virus resistance with only a few small damage points present on the leaves; 5 of them were scored Level 3 or 2 and showed reduced virus damage; and 3 transgenic lines showed susceptibility to SCMV infection that was similar to non-transgenic control plants (TC and WT) and obvious virus damage. Compared to non-transgenic control sugarcane lines, the transgenic sugarcane plants showed enhanced resistance to SCMV infection by representative SCMV isolates. The trial was repeated three times and similar results were obtained (Table 2).

**Table 1:** **Summary of regeneration and transformation of transgenic sugarcane lines**.

**Sugarcane variety**	**Bombarded calli**	**Kanamycin resistant calli**	**Kanamycin resistant lines**	**Southern blotting positive lines**
Badila	2251	174	53	13

**Table 2:** **Summary of reactions to artificial inoculation in vegetatively propagated transgenic sugarcane progeny under greenhouse conditions**.

**Lines**	**No. of plants inoculated**	**Susceptible (No. of plants)**	**Recovery (No. of plants)**	**Resistant (No. of plants)**	**Resistance level**	**Kanamycin assay (No. of plants)**	**Selectable marker expression^a^ (% of plants)**	**Transgene copy number**
B_1_	20	2	2	16	MR	10	100	3
B_2_	20	0	3	17	HR	10	100	1
B_4_	20	12	3	5	S	10	100	2
B_12_	20	5	4	11	R	10	100	1
B_15_	20	3	3	14	MR	10	100	3
B_19_	20	10	3	7	S	10	100	2
B_23_	20	4	2	14	R	10	100	1
B_31_	20	13	1	6	S	10	100	2
B_36_	20	0	1	19	HR	10	100	1
B_38_	20	0	2	18	HR	10	100	1
B_42_	20	3	5	12	MR	10	100	1
B_48_	20	0	0	20	I	10	100	1
B_51_	20	0	3	17	HR	10	100	1
WP (CK_1_)	20	18	2	0	HS	10	0	0
TC (CK_2_)	20	8	12	0	S	10	0	0

B1–B51, Different transgenic sugarcane lines; TC, untransformed tissue culture events; WP, wild parental clone of sugarcane plants (non-transgenic and non-tissue culture derived); HS, highly susceptible; S, susceptible; R, resistant; MR, moderately resistant; HR, highly resistant; I, immune.

a *Plants not expressing npt II gene exhibited leaf chlorosis in the field following spraying with a kanamycin solution (3.0 g/L)*.

### Stability of resistance to SCMV infection in transgenic sugarcane plants under field conditions

To evaluate the stability of resistance to SCMV infection in transgenic sugarcane lines grown under natural field conditions, field virus trials were conducted followed by artificial inoculation and natural aphid transmission. Five selected independent transgenic sugarcane lines (B_2_, B_36_, B_38_, B_48_, and B_51_), which had a better performance against SCMV under the greenhouse conditions, one TC event, and one WT line, from the first to fourth vegetative propagated generation, respectively, were selected for the field trials conducted at four different experimental field sites in China (Table 3).

**Table 3:** **Disease incidence in vegetatively propagated transgenic sugarcane progeny and control sugarcane plants under field conditions**.

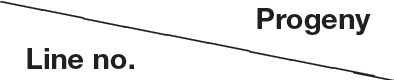	**T_1_**	**T_2_**	**T_3_**	**T_4_**	**Resistance level**	**Selectable marker expression^a^(% of plants)**
B_2_	12.3	12.1	12	20.1	MR	100
B_36_	6.4	5.6	5.4	21.6	R	100
B_38_	24.0	28.9	16.2	26.5	MR	100
B_48_	0.0	0.0	0.0	2.64	HR	100
B_51_	19.7	18.4	8.0	16.8	MR	100
WT (CK_1_)	91.7	92.1	100	98.4	HS	0
TC (CK_2_)	50.0	41.0	31.6	43.8	S	0

B2, B36, B38, B48, and B51, Different transgenic sugarcane lines; TC, untransformed tissue culture events; WP, wild parental clone of sugarcane plants; HS, highly susceptible; S, susceptible; R, resistant; MR, moderately resistant; HR, highly resistant; I, immune.

a *Plants not expressing NPTII gene exhibited leaf chlorosis in the field following spraying with a kanamycin solution (3.0 g/L)*.

In the artificial inoculation test under field conditions, the disease incidence of untransformed parent clones (WT) of Badila was more than 90%. For non-transgenic tissue culture clones, the disease incidence was ~40%. Typical symptoms of SCMV infection, such as vein clearing and mottled leaves, were commonly observed in non-transgenic control plants (TC and WT). The incidence of SCMV infection was greatly reduced in the clones obtained by tissue culture relative to the parental clones. These results are consistent with previous reports of reduced SCMV infection using apical meristem culture (Ramgareeb et al., 2010). The disease incidence of transgenic sugarcane lines was less than 25%, suggesting that SCMV CP expression resulted in improved resistance against SCMV in transgenic sugarcane plants under field conditions.

Field virus resistance of these transgenic plants to SCMV infection was successfully demonstrated in field trials and the infection rates were nearly the same across different generations (Table 3), indicating that the protection against SCMV infection conferred by the SCMV-CP transgene was stably transmitted to the progeny by vegetative propagation in the transgenic sugarcane lines.

### Expression analysis of the selectable marker *nptII* gene

The expression of antibiotic resistance mediated by the selectable marker *nptII* gene was verified by a kanamycin field assay. All transgenic sugarcane plants with the *nptII* gene grown both under field conditions and in greenhouses displayed 100% resistance to kanamycin (Tables 2, 3). In contrast, non-transgenic parental clones of sugarcane plants (WT) and untransformed tissue culture (TC) samples without the *nptII* gene showed leaf chlorosis symptoms on all sugarcane plants sprayed with 3.0 g/L kanamycin.

### Comparison of growth rates under field conditions

**Figure 2:**
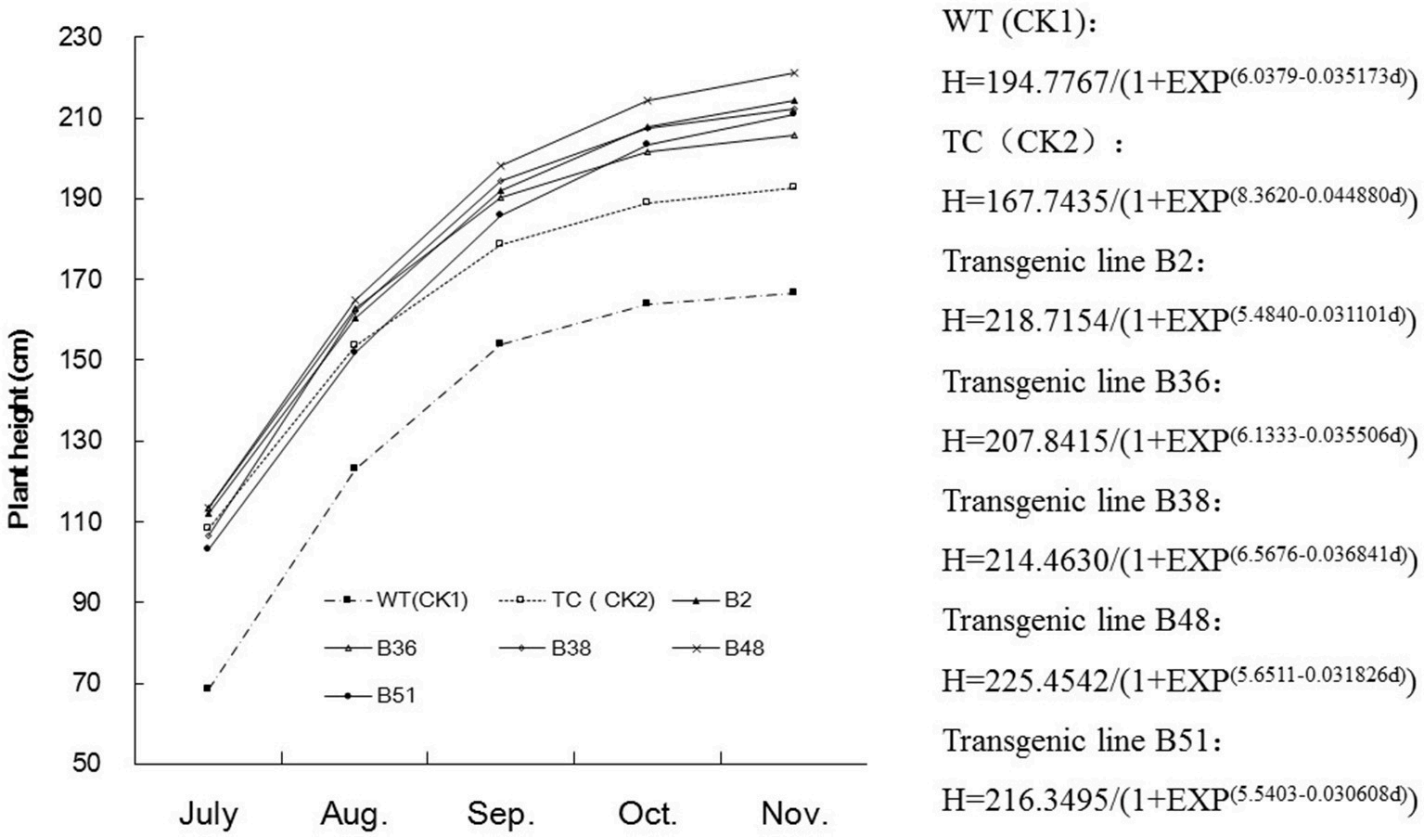
**Comparison of growth curve of sugarcane stalk in different transgenic lines**. B_2_, B_36_, B_38_, B_48_, and B_51_, Different transgenic sugarcane lines; TC (CK_2_), untransformed tissue culture events; WT (CK_1_), wild-type parental clone of sugarcane plants.

A comparison of growth rates showed that the transgenic sugarcane lines were more vigorous than the control sugarcane plants (WT and TC) under field conditions. The transgenic sugarcane plants grew faster and reached more than one meter in height by early July, which was 30 cm higher than the height of the control sugarcane plants (WT and TC). During the elongation stage, the transgenic sugarcane plants grew faster than the control plants, as shown by plotting a logistic growth curve (Figure 2). Taken together, these results confirmed that virus infection can significantly reduce the growth rate of sugarcane and eventually decrease stalk yields at harvest.

### Agronomic and yield trait assessment under field conditions

**Figure 3:**
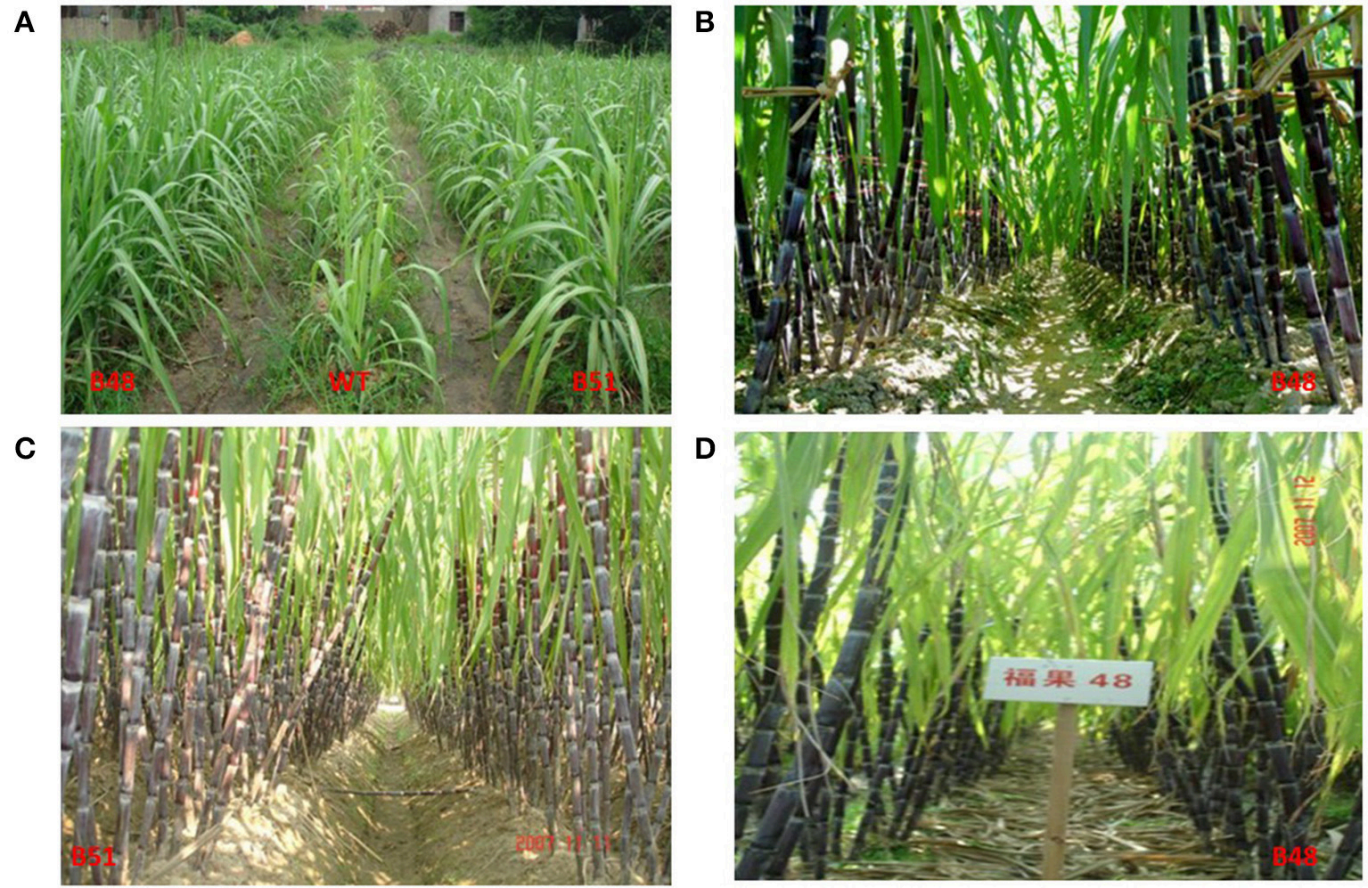
**Field performance of transgenic lines in different experimental locations**. **(A)** B48 in Fuzhou, Fujian, photo taken on May 6, 2007; **(B)** B48 in Jianyang, Fujian, photo taken on Nov. 4, 2007; **(C)** B51 in Wenling, Zhejiang, photo taken on Nov. 11, 2007; **(D)** B48 in Yiwu, Zhejiang, photo taken on Nov. 12, 2007.

Compared to the parent clone (Badila, WT), all 5 transgenic lines (B38, B36, B48, B51, and B2) produced higher Brix, greater cane yield, longer internode and stalk, more millable stalk, and smaller stalk. In addition, single stalk weight was also higher in the 4 transgenic lines except for B36. The mean cane yield of the 5 transgenic lines and their wild-type parental Badila ranged from 61.61 t/ha (Badila) to 102.72 t/ha (B48) and the highest cane yield was obtained from transgenic B48 (Brix: 16.60 vs. 15.25%, respectively; Table 4). The analysis of stability showed that Badila (WT) was the most unstable as determined by W_i_ and ${\text{S}}_{\text{d}}^{2}$ for the traits of cane yield, stalk height, internode length, millable stalk, stalk weight, and Brix. Linear regression for each trait of a single genotype on all sugarcane lines in each environment resulted in regression coefficients (b values) ranging from 0.45 to 1.42 for cane yield and 0.03 to 1.58 for Brix. This large variation in regression coefficients indicated different responses of the sugarcane lines to environmental changes (Table 4). Transgenic B48 had better performance in good environments with the largest regression coefficients for cane yield, stalk height, single stalk weight, and Brix. In contrast, Badila (WT) had the smallest regression coefficients for cane yield and Brix and the worst performance went grown in good environments. WT grew very slowly at the early growth stage with blotchy mottle leaves that were infected by SCMV virus (Figure 3). The regression coefficients of B48 for internode length, millable stalk number, and stalk diameter were close to 1.0 and had a small deviation from regression (${\text{S}}_{\text{d}}^{2}$). Therefore, B48 exhibited fair stability for these traits.

**Table 4:** **Mean performance, regression (bi), and deviation from regression of agronomic traits for five transgenic sugarcane lines evaluated at four locations using the Eberhart and Russell model**.

**Traits**	**Transgenic lines**	**Mean performance**	**Effect**	**Variance (W_i_)**	**Coefficient variation (${\text{S}}_{\text{d}}^{2}$)**	**Regression coefficient (b)**
Cane yield (t/ha)	B_48_	102.72 ± 22.73^a^	19.43	82.016	8.82	1.425
	B_51_	89.91 ± 21.73^ab^	6.62	116.511	12.00	1.721
	B_38_	85.49 ± 22.92^ab^	2.20	65.649	9.48	0.449
	B_2_	84.02 ± 27.42^ab^	0.73	3.942	2.36	1.021
	B_36_	76.28 ± 27.70^bc^	–7.01	10.771	4.30	0.824
	WT	61.31 ± 15.05^c^	–21.98	144.296	19.59	0.560
Internode length (cm)	B48	9.56 ± 0.427^a^	0.479	0.122	3.648	0.933
	B38	9.33 ± 0.612^a^	0.249	0.089	3.191	2.086
	B36	9.33 ± 0.627^a^	0.246	0.333	6.192	–1.107
	B2	9.22 ± 0.315^a^	0.136	0.048	2.368	0.499
	B51	9.13 ± 0.762^a^	0.054	0.146	4.180	1.349
	WT	7.91 ± 1.404^b^	–1.166	0.194	5.571	2.240
Stalk height (cm)	B_48_	227.5 ± 21.81^a^	13.27	37.55	2.69	1.539
	B_36_	221.7 ± 27.64^a^	7.50	4.72	0.98	0.795
	B_51_	221.2 ± 17.80^a^	6.93	2.67	0.74	0.985
	B_2_	220.6 ± 23.72^a^	6.35	24.21	2.23	0.737
	B_38_	218.1 ± 23.88^a^	3.85	4.32	0.95	1.123
	WT	176.3 ± 30.26^b^	–37.9	42.22	3.68	0.821
Millable stalk number (× 10^4^/ha)	B48	5.713 ± 1.006^a^	0.639	0.030	3.05	0.96
	B51	5.419 ± 1.446^a^	0.345	0.193	8.11	1.65
	B38	5.238 ± 1.428^a^	0.163	0.088	5.65	1.42
	B2	5.192 ± 0.934^a^	0.117	0.004	1.24	1.10
	B36	5.043 ± 1.092^a^	–0.031	0.045	4.20	1.30
	WT	3.842 ± 0.533^b^	–1.233	0.927	25.06	–0.43
Single stalk weight (kg)	B48	1.784 ± 0.374^a^	0.162	0.010	5.69	1.42
	B51	1.645 ± 0.260^a^	0.023	0.006	4.83	1.04
	B38	1.634 ± 0.215^a^	0.011	0.012	6.63	0.68
	B2	1.607 ± 0.277^a^	–0.015	0.000	1.06	0.94
	WT	1.560 ± 0.290^a^	–0.062	0.007	5.41	0.90
	B36	1.503 ± 0.269^a^	–0.119	0.002	2.80	1.02
Stalk diameter (cm)	WT	3.604 ± 0.264^a^	0.270	0.001	0.839	1.061
	B_48_	3.372 ± 0.211^b^	0.038	0.001	1.006	1.205
	B_38_	3.312 ± 0.147^bc^	–0.022	0.012	3.257	0.424
	B_51_	3.306 ± 0.206^bc^	–0.028	0.004	1.821	1.015
	B_2_	3.262 ± 0.242^bc^	–0.072	0.001	0.782	1.127
	B_36_	3.147 ± 0.263^c^	–0.186	0.004	1.954	1.169
Brix (%)	B48	16.60 ± 2.16^a^	0.529	0.113	2.027	1.355
	B36	16.33 ± 2.12^ab^	0.265	0.018	0.833	1.117
	B38	16.18 ± 2.00^ab^	0.112	0.039	1.223	1.087
	B2	16.06 ± 2.23^ab^	–0.010	0.066	1.598	0.836
	B51	15.99 ± 2.34^ab^	–0.081	0.117	2.138	1.579
	WT	15.25 ± 1.52^b^	–0.815	0.423	4.266	0.026

*B2, B36, B38, B48, and B51, Different transgenic sugarcane lines; WT, wild parental clone of sugarcane plants (Badila). The same letter showed no significant differences at the level of 0.05*.

The industrial relevant sugarcane traits pertaining to sugar quality, such as sucrose in juice, gravity purity, and Brix, showed no obvious difference between the control plants (WT and TC) and transgenic plants under the field conditions (Table 5). Therefore, these results indicated that the damage caused by virus infection in the control plants affected sugar quantity but not quality.

**Table 5:** **Industrial traits of transgenic lines in field trials**.

**Transgenic lines**	**Sucrose in cane (%)**	**Brix in juice (%)**	**Sucrose in juice (%)**	**Gravity purity (%)**	**Fiber in cane (%)**
B_2_	14.32	19.7	44.54	44.54	11.56
B_36_	14.16	20.1	44.57	44.57	9.88
B_38_	13.41	19.0	43.97	43.97	9.72
B_48_	14.96	20.2	45.36	45.36	10.06
B_51_	14.55	19.7	44.89	44.89	10.06
WT	13.34	18.6	43.40	43.40	8.68

*B2, B36, B38, B48, and B51: Different transgenic sugarcane lines; WT, wild parental clone*.

## Discussion

The agronomic performance and disease resistance evaluated in our field trials over two successive seasons at four different experimental field locations indicated the superiority of transgenic plants of Badila expressing the *CP* gene from the SCMV-D strain. Our results demonstrated that the transgenic sugarcane line B48 had an average of 102.72 tons of cane per acre per year, which was almost two-fold higher than that of control sugarcane plants (Badila). In addition, the SCMV infection rate in transgenic lines B48 was less than 3% over the past 5years compared to 91–100% in wild-type parental plants (Badila, WT). Badila is one of the most popular cultivars that are grown for table cane in China due to its ability to produce abundant juice, less fiber, and good flavor. Badila is also the most important noble parent in the sugarcane genetic improvement program. Unfortunately, Badila is highly susceptible to the SCMV virus with more than 95% disease incidence. Transgenic plants expressing the SCMV-CP gene generated from Badila had enhanced cane yield (TCH) and sugar content (TSH) as well as lower SCMV disease incidence. Most of the control plants developed virus infection symptoms that persisted and spread throughout the plant with more than 30% incidence in the tissue plant (TC) and over 90% in Badila (WT). The SCMV infection incidence was lower in clones derived from tissue cultures compared to WT, which is consistent with previous reports of SCMV declination through apical meristem culture (Ramgareeb et al., 2010). Although apical meristem tissue culture would be simpler to perform than genetic transformation, sugarcane derived from tissue culture can be easily re-infected upon subsequent exposure to the SCMV virus (Cheong et al., 2012).

SCMV is one of the most threatening viral pathogens of sugarcane worldwide. Pathogen-derived resistance (PDR) was first conceptualized in interactions between plant hosts and their pathogens, wherein integration of parts of the virus genome into the host can confer virus resistance. PDR was first demonstrated with plants transformed with the CP gene of tobacco mosaic virus (TMV) that were resistant to subsequent infection by TMV (Abel et al., 1986). Since then, numerous reports of PDR genes that confer resistance to a wide range of virus-host combinations have been reported (Baulcombe, 1996; Grumet, 2010). The first transgenic squash (Freedom II) with resistance to zucchini yellow mosaic virus was released commercially in the USA (Alshahwan et al., 1995). The use of pathogen-derived genes to confer resistance was also exploited as a strategy to develop SCMV-resistant sugarcane via genetic engineering. The CP is the structural component of the virus particle and is composed of repeating CP subunits that help protect the viral genome. The SCMV CP has been previously cloned (Shukla and Ward, 1988; Yao et al., 2006) and developed into a construct that was suitable for expression of the gene in sugarcane plants (Smith et al., 1992). Several independent SCMV-CP transgenic sugarcane plants have been reported and their field performance has been evaluated in Australia (Smith et al., 1992) and the United States (Gilbert et al., 2005). Our results indicated that the transgenic line B48 was “immune” to SCMV infection in greenhouse and field tests as evidenced by the lack of SCMV symptoms and virus as analyzed by ELISA and RNA-sequencing (data unshown). These traits were preserved over the course of several years. Virus immunity is one phenotype that has been described for other transgenic plant systems (Smith et al., 2000) and is the most obvious form of transgene-mediated virus resistance. However, the precise mechanism by which transgenes mediate virus resistance, and whether they act at an RNA or protein level in plants, is currently unknown. The consistency and stability of transgenic expression are important traits for future commercial applications of transgenic sugarcane. The results reported here are particularly significant in that they provide a rigorous, objective analysis of several transgenic lines compared to control sugarcane lines under field conditions. Although Dale and McPartlan (1992) found that GUS expression had a significant negative effect in potato, *nptII* expression did not affect agronomic and yield performance, indicating that *nptII* does not affect primary metabolism of the plants and thus transgenic plants can be compared to wild-type counterparts under similar growth conditions. In this study, kanamycin assays verified that the selective marker gene *nptII* was stably expressed in all transgenic sugarcane lines, which supports that this assay is a simple and effective approach to verify the presence of *nptII* selective marker gene expression under field conditions. In summary, our field evaluation results confirmed the potential of genetic transformation methods to integrate disease resistance traits into sugarcane. The transgenic line B48 can be used as a parent to combine SCMV resistance with the agronomic merits of high-yield sugarcanes in a sugarcane breeding program. The use of the transgenic line B48 with its SCMV resistance is likely to be a useful option for use in traditional breeding approaches, particularly given that SCMV resistance in sugarcane is limited in naturally occurring sugarcane populations.

## Author contributions

MZ and RC conceived and designed the experimental plan. WY, MR, CY, and LQ performed the experiments. WY analyzed the data and wrote the paper. MZ, RC, and BC revised the paper. All authors read and approved the final version of the paper.

### Conflict of interest statement

The authors declare that the research was conducted in the absence of any commercial or financial relationships that could be construed as a potential conflict of interest.
